# Promoting Therapists’ Use of Motor Learning Strategies within Virtual Reality-Based Stroke Rehabilitation

**DOI:** 10.1371/journal.pone.0168311

**Published:** 2016-12-19

**Authors:** Danielle E. Levac, Stephanie M. N. Glegg, Heidi Sveistrup, Heather Colquhoun, Patricia Miller, Hillel Finestone, Vincent DePaul, Jocelyn E. Harris, Diana Velikonja

**Affiliations:** 1 Department of Physical Therapy, Movement Sciences and Rehabilitation, Bouve College of Health Sciences, Northeastern University, Boston, Massachusetts, United States of America; 2 Therapy Department, Sunny Hill Health Centre for Children, Vancouver, British Columbia, Canada; 3 School of Rehabilitation Sciences, Faculty of Health Sciences, University of Ottawa, Ottawa, Ontario, Canada; 4 Department of Occupational Science and Occupational Therapy, University of Toronto, Toronto, Ontario, Canada; 5 School of Rehabilitation Sciences, Faculty of Health Sciences, McMaster University, Hamilton, Ontario, Canada; 6 Physiatry, Élisabeth Bruyère Hospital and Bruyère Continuing Care, Ottawa, Ontario, Canada; 7 School of Rehabilitation Therapy, Queen's University, Kingston, Ontario, Canada; 8 Regional Rehabilitation Center, Hamilton Health Sciences, Hamilton, Ontario, Canada; Purdue University, UNITED STATES

## Abstract

**Purpose:**

Therapists use motor learning strategies (MLSs) to structure practice conditions within stroke rehabilitation. Virtual reality (VR)-based rehabilitation is an MLS-oriented stroke intervention, yet little support exists to assist therapists in integrating MLSs with VR system use.

**Method:**

A pre-post design evaluated a knowledge translation (KT) intervention incorporating interactive e-learning and practice, in which 11 therapists learned how to integrate MLSs within VR-based therapy. Self-report and observer-rated outcome measures evaluated therapists’ confidence, clinical reasoning and behaviour with respect to MLS use. A focus group captured therapists’ perspectives on MLS use during VR-based therapy provision.

**Results:**

The intervention improved self-reported confidence about MLS use as measured by confidence ratings (p <0.001). Chart-Stimulated Recall indicated a moderate level of competency in therapists’ clinical reasoning about MLSs following the intervention, with no changes following additional opportunities to use VR (p = .944). On the Motor Learning Strategy Rating Instrument, no behaviour change with respect to MLS use was noted (p = 0.092). Therapists favoured the strategy of transferring skills from VR to real-life tasks over employing a more comprehensive MLS approach.

**Conclusion:**

The KT intervention improved therapists’ confidence but did not have an effect on clinical reasoning or behaviour with regard to MLS use during VR-based therapy.

## Introduction

Best-practice rehabilitation interventions after stroke should incorporate practice conditions known to promote the neuroplastic processes underlying motor (re)learning [1]. Therapists can structure these practice conditions through their use of motor learning strategies (MLS): observable therapeutic actions in which therapists consider task and client-specific factors to select and to apply evidence-based practice and feedback variables for optimal motor learning [2]. In the acute and subacute stages of stroke recovery, therapists can use MLS to provide augmented feedback and to structure the abundant practice of motivating, meaningful tasks within an enriched environment [3,4]. The goal of MLS application is to support a client’s ability to retain and to transfer the learning achieved in therapy to improved performance in a real world context. Although motor learning is recognized as a key foundation for neurological physical therapy approaches [5,6], it can be challenging for therapists to determine the specific MLS most relevant to their individual clients [7]. The abundance of MLS options, the inability of experts to come to consensus about MLS definitions and use [8] and the limited educational resources to support therapists in their use [7] all limit MLS application in clinical practice.

Virtual reality (VR), defined as any computer hardware and software system that generates simulations of real or imagined environments with which participants interact using body movements [9], is an increasingly popular treatment modality in stroke rehabilitation, with evidence building to support its use [10–12]. While VR systems differ in their interaction methods (e.g. holding a remote input device, using motion-capture technology), they share common attributes, including offering goal-oriented tasks and abundant visual and auditory feedback [13–15]. These attributes may enhance client motivation to participate in therapy interventions [16,17], which is posited to enhance motor learning. Given the differences in somatosensory input and extrinsic feedback between a VR-based approach in a virtual environment, and a conventional rehabilitation approach in a physical environment [18–20], therapists may question whether and how to integrate MLS into their VR sessions [21,22].

Therapists who provide VR-based therapy must make decisions about appropriate games that match individual client capabilities and treatment goals [23,24]. This decision-making can be complex and is founded in understanding VR system operation and game play requirements. Knowledge translation (KT) interventions that combine training on these foundational skills with MLS knowledge may help therapists take advantage of the motor learning attributes offered by the VR system. Examples of these attributes include feedback about results in the form of a score, or feedback about performance when clients can observe their own movements in the virtual environment. Such KT interventions can also target a therapist’s ability to optimally adjust VR system software parameters (e.g. to promote a more variable practice schedule) and to promote transfer of learning of skills practiced in the virtual environment to the real world [23].

KT interventions, such as interactive education involving a clinical manual and clinical and technical support, have been shown to improve knowledge and skills about use of VR in practice [25–27]. However, eliciting actual behavior changes through KT interventions is more challenging [28]. Evidence supports multi-faceted KT strategies that are tailored to address specific barriers to behavior change [29–31]. Defined as “e-learning products that translate evidence-based knowledge to disseminate information that increases awareness, informs clinical practice and/or stimulates practice change” [32] p. 649, online KT resources are attractive because of their potential to provide multimedia content that can be accessed at an individual’s own pace [33]. In combination with experiential practice focusing on hands-on skills in VR system use and in MLS application, online KT resources may be an ideal way to translate knowledge about MLS. Indeed, 63% of Canadian physiotherapist respondents of a survey on motor learning principle application in neurorehabilitation identified online modules as their preferred educational format [7].

The purpose of this study was to develop and to evaluate the effectiveness of a multi-faceted KT intervention designed to support the use of a motor learning approach to VR-based therapy by physical therapists (PTs) and occupational therapists (OTs) in stroke rehabilitation. Levac et al. [26] report findings from the current study related to changes in therapists’ knowledge and self-reported skills related to VR system use. The current study was designed to address the following question: Does participation in a KT intervention influence therapists’ confidence, clinical reasoning, and observed application of MLS during VR interventions? We hypothesized that therapists’ clinical reasoning about, and observable demonstration of MLS would increase between time 1 (following the KT intervention and initial use of the VR system with clients) and time 2 (following additional opportunities to use VR in their clinical practice).

## Materials and Methods

A pre-post design was used to evaluate a KT intervention at two sites with measures post-initial training (time 1) and after additional practice opportunities (time 2).

### Procedures

The study was carried out at two rehabilitation sites using a pre-post design where the KT intervention was evaluated with specific measures assessing clinical reasoning and MLS that were collected after initial training (Time 1) and following additional practice opportunities (Time 2).

PTs and OTs working part- or full-time were recruited from inpatient and outpatient stroke rehabilitation units at two rehabilitation centres in Ontario, Canada. Both sites already had a VR system in place but the therapists had not received any training in its use. Ethics approval was obtained from the Hamilton Health Sciences Research Ethics Board (Project # 13–384), the University of Ottawa Research Ethics Board (Protocol # A12-12-03) and the Bruyere Continuing Care Research Ethics Board (Protocol #M16-12-038). Therapist and patient participants provided written informed consent to participate, the procedure for which was approved by each Research Ethics Board.

At each site, the KT intervention was delivered over a period of 5 months with a staggered start time between sites. During the first month, therapists completed the e-learning modules outside of working hours at their own pace. They then took part in group and individual hands-on learning sessions during working hours. Over the next four months, each therapist identified up to four patients who 1) had experienced a stroke within the last year; 2) were receiving inpatient or outpatient PT and/or OT services focused on improving motor skills; and 3) were judged by the therapist to have appropriate cognitive and physical skills to engage with the VR system. No specific inclusion or exclusion criteria were provided. A research assistant (RA) recruited patients into the study. Therapists then implemented up to four sessions of VR-based therapy per patient. Time 1 outcome data collection occurred after the first two clients and Time 2 outcome data collection occurred after the next 2 clients. One session for every second patient was videotaped for outcome measurement purposes. The taped session was the final session with the second client at each time point (i.e. Client 2 and 4; or, if only 2 clients were recruited, Client 1 and 2). This process was carried out in order to evaluate any changes in MLS use following additional experience. The RA recorded the number of clients who had been identified by therapists as suitable for participation in the study as well as the number who had been approached for recruitment but declined to participate. Finally, Site 2 therapists were offered the opportunity to interact with clinicians at Site 1 to obtain mentoring support via email, Skype or phone contact. Other than the enhanced mentorship at Site 2, the KT interventions employed at each site were the same. Our interest was in determining whether enhanced mentorship by therapists who had just undergone the training would be of added benefit to the mentees and the mentors.

**Fig 1:**
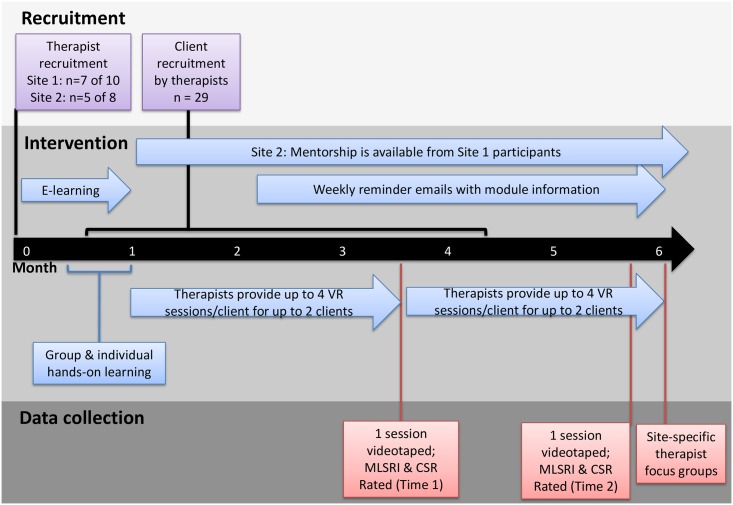
Study procedures.

Fig 1 illustrates the study procedures.

### Virtual reality system

The GestureTek Interactive Rehabilitation Exercise System (IREX; www.gesturetekhealth.com, GestureTek, Toronto, ON, Canada) is a video-capture VR system in which clients view a mirror image of themselves interacting with virtual objects. At the time of the study, the GestureTek system was a prominent rehabilitation-specific VR system, possessing the greatest volume of promising evidence, varying from low to strong quality, for upper extremity movement quality, dynamic balance and locomotor function outcomes in adults after stroke [34].

### Intervention

The following overview of the five components of the KT intervention has been structured according to the Template for Intervention Description and Replication (TIDieR) checklist guide for describing interventions [35].

1. E-learning modules: In addition to one e-learning module instructing therapists on the use of the VR system and its games [26], two e-learning modules presented information about neuroplasticity, MLS, motor learning attributes of VR systems, and implementation of MLS into VR-based therapy. Examples of learning objectives included the ability to provide examples of using motor learning principles in practice, to describe how to link skills practiced in VR to real-life skills or activities, and to list ways to incorporate variable practice into VR-based therapy. Therapists completed each e-learning module in approximately two hours and received compensation for this time. Therapists were also provided with a printed manual containing the information presented in the online modules.
2. Hands-on learning: One individual and two group hour-long sessions provided by the PI (DL) and a clinician (MB) enabled hands-on experience using the IREX and facilitated discussion and practice of MLS using case scenarios. Feedback about MLS use during VR-based therapy was provided to participants by the PI (DL) during the individual practice sessions.
3. Experiential learning: Clinicians then used the IREX with up to four of their clients with stroke who agreed to participate in the study. This use was not supervised by the research team, and no feedback was provided, although therapists were free to request feedback from the study investigators.
4. Didactic reminders: During the experiential learning phase, weekly reminder emails were sent by the PI to therapists, which contained knowledge from the modules as well as motivational tips and tricks for IREX use.
5. Mentorship: No specific mentorship opportunities were facilitated at Site 1. Clinicians at Site 2 were matched with one of three clinician mentors from Site 1 who had recently completed the KT intervention. Site 2 clinicians received biweekly encouragement to contact mentors with any questions.

### Outcomes

#### Confidence

Confidence ratings: Therapists rated their confidence with respect to each learning objective before and after completing each e-learning module and at study completion using a seven-point Likert scale. The time between completion of pre- and post-module confidence logs varied from hours to days, depending on how long therapists spent completing the module (this was not recorded).

#### Clinical Reasoning

Chart-Stimulated Recall (CSR) was utilized to assess clinical reasoning related to VR and MLS use. CSR involves a semi-structured interview administered to therapists by trained interviewers [36]. The interview “engages participants in a reflective discussion on these deeper levels of cognitive reasoning” [37]. Study investigators determined nine competencies of interest (listed in table 1) and developed seven-point Likert-scale indicators for each competency. Table 2 provides an example of grading criteria for competency 4, in which the rater asks the question “Can you tell me about some of the things that you did to get the right fit in terms of the difficulty of the VR-based therapy in the videotaped session?” Therapists brought their PT/OT session documentation notes and began by watching a video of themselves carrying out a therapy session using IREX with a client. Through guided questioning, therapists were asked to share their clinical reasoning while describing their use of MLS during the session. The nine competencies were scored by two independent raters who had undergone a standardized training process, including a practice interview. Following each interview, the raters discussed and achieved consensus about a final score for each of the nine competencies. CSR total score is a percentage of total points in each competency divided by the maximum total score of 63 points.

**Table 1:** Chart-stimulated recall competencies.

Chart-Stimulated Recall Motor Learning Competency
Decision-making for initial VR treatment program parameters Part 1 –Getting started
Decision-making for initial VR treatment program parameters Part 2 –Adjusting parameters
Using VR to target overall therapeutic goals
Grading to make practice challenging
Practice is meaningful
Practice is task-oriented
The client is as an active problem-solver
The therapist provides feedback
Evaluation of outcomes of VR treatment

**Table 2:** Example of chart-stimulated recall grading criteria for competency 4.

Likert scale	Criteria	PRE consensus	POST consensus
1	Therapist cannot describe any strategies used to achieve a ‘just-right’ challenge in the videotaped session.		
2	Therapist can describe the importance of achieving a ‘just-right’ challenge from a motor learning perspective but cannot describe any strategies used in the videotaped session.		
3	Therapist describes one strategy of adjusting the difficulty level and/or duration setting of the games.		
4	Therapist provides one example of a strategy used to achieve a ‘just-right’ physical OR cognitive challenge		
5	Therapist provides two examples of strategies used to achieve a ‘just-right’ physical OR cognitive challenge		
6	Therapist provides one example of a strategy used to achieve a ‘just-right’ physical challenge AND one example of a strategy used to achieve a ‘just-right’ cognitive challenge.		
7	Therapist provides two examples of strategies used to achieve a ‘just-right’ physical challenge AND two examples of strategies used to achieve a ‘just-right’ cognitive challenge. OR, therapist provides a sound rationale (including both physical and cognitive concepts) as to why the fit was already right.		

#### Behaviour

Motor Learning Strategy Rating Instrument (MLSRI-20): The MLSRI-20 is a 20-item observer rating scale that uses a five-point Likert scale to evaluate how therapists implement MLS during treatment sessions [2,38]. Initial validation and reliability work in a sample of therapists working with children with acquired brain injury (ABI) demonstrated strong psychometric promise [38] but pointed to the need to create a shorter version and to clarify certain items to facilitate its use by clinicians. These changes were informed through a process involving international researchers which evaluated face validity [39]. Inter-rater reliability evaluation of the new MLSRI-20 with therapists working with children with cerebral palsy and ABI yielded an ICC of 0.79 [39]. The MLSRI-20 is a 4 point Likert Scale (0 not observed and 4 observed to a very great extent) in which a trained rater watches a videotaped treatment session and scores the use of MLSs on 20 items in three categories: What Therapist Says, What Therapist Does, and What Practice Involves. MLSRI-20 item number 19 (practice is random, rather than blocked) in the ‘Practice Involves’ category was omitted for this study because it could not be rated in the context of VR interventions. Higher scores are associated with greater MLS frequency.

### Focus groups

The principal investigator (PI) conducted a 1.5-hour focus group at each site at the end of the study to explore therapists’ perspectives on the KT intervention, their use of the IREX in practice (reported in [26]) and their experience of integrating MLS use into VR-based therapy. The focus group was audio-recorded and transcribed for analysis.

### Analyses

Non-parametric Wilcoxon signed rank tests were used to evaluate MLSRI-20 and CSR scores and confidence ratings. Distribution normality of ordinal (treated as interval) data was evaluated using plots. Descriptive statistics summarize confidence ratings, MLSRI items and CSR competencies at each time point. Qualitative content analysis [40] was used to categorize responses to the open-ended questions. Two investigators (DL and PM) independently reviewed focus group transcripts and met to come to consensus on categories.

## Results

All therapists completed the confidence ratings pre-module, post-module and post-study. All 11 completed the CSR and MLSRI-20 at Time 1, but only 8 completed these measures at Time 2, because recruitment issues prevented a second intervention session from being videotaped for the other three therapists. Histogram plots, skewness and kurtosis values demonstrated acceptable evidence of normality for all outcome measures at each time point.

### Participant demographics and intervention fidelity

Therapist and client demographics are summarized in table 3. All therapists were novice IREX users. Table 4 summarizes the number of clients recruited by therapists at each site. There was no uptake of the mentoring offered by Site 1 clinicians to Site 2 clinicians. As such, outcome measure results for both sites are presented together since there was no difference between the interventions at the sites. Technical issues with the IREX at each site influenced intervention fidelity, as a period of several weeks passed during the study at each site during which the system was non-functioning and awaiting repair.

**Table 3:** Participant and client demographics.

Site	Profession	Clinical experience	Total # of clients	# of clients recruited per therapist	Client mean age	Mean client length of stay
Ottawa	4 OTs, 2 PTs	19.3 (SD 8.1) yrs	24	3 therapists: 4 clients 1 therapist: 3 clients, 2 therapists: 2 clients	62.8 (SD 16.4) yrs	123.8 (SD 166.7) days, range 21–682
Hamilton	3 OTs, 2 PTs	11.4 (SD 9.4) yrs	15	2 therapists: 4 clients 1 therapist: 3 clients 2 therapists: 2 clients	60.1 (SD 15.0) yrs	131.4 (SD 176.7) days, range 14–624

OT = occupational therapist; PT = physical therapist; SD = standard deviation; yrs = years

**Table 4:** Chart-stimulated competency ratings at Time 1 and Time 2.

Chart-Stimulated Recall Motor Learning Competency	Mean (SD) Time 1	Mean (SD) Time 2	P value
Decision-making for initial VR treatment program parameters Part 1 –Getting started	5.4 (1.6)	5.5 (1.2)	0.866
Decision-making for initial VR treatment program parameters Part 2 –Adjusting parameters	4.0 (2.2)	2.8 (2.2)	0.206
Using VR to target overall therapeutic goals	4.9 (1.9)	4.6 (2.2)	0.944
Grading to make practice challenging	4.7 (0.9)	4.3 (1.3)	0.414
Practice is meaningful	2.5 (2.2)	3.3 (2.6)	0.066
Practice is task-oriented	3.6 (2.3)	3.5 (2.5)	0.734
The client is as an active problem-solver	3 (1.8)	2.6 (1.4)	0.492
The therapist provides feedback	3.2 (2.1)	3 (2.7)	0.916
Evaluation of outcomes of VR treatment	3.9 (1.9)	3.6 (2.1)	0.435
Total score	35.2 (11.1)	33.1 (11.6)	0.944

### Confidence

**Fig 2:**
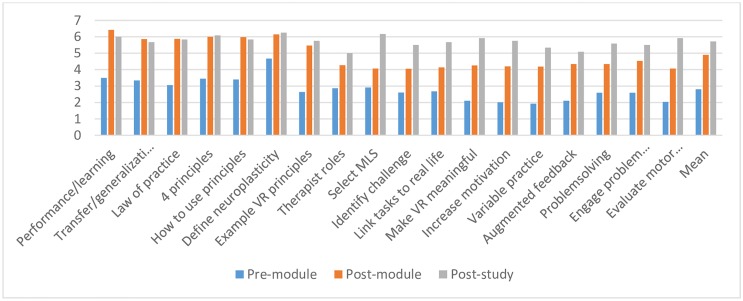
Pre module, post module and post study confidence ratings.

The mean improvement in confidence ratings from pre- to post-module completion was 2.09 (SD 0.55) points; Z = -3.724, p < 0.01], with a further mean increase at study end of 0.81 (SD 0.88) points. Fig 2 illustrates mean confidence scores on each learning objective at each time point.

### Clinical reasoning

Eight of the 11 therapists completed the CSR at both time points. CSR total scores (55.9% at time 1 and 52.6% at time 2) indicate a moderate level of competency in therapist decision-making about MLS use; no significant difference between time points was observed in total (p = 0.944) or item scores. Competency related to getting started with a VR intervention program was rated highest (mean 5.4 at Time 1, 5.5 at Time 2). Articulating strategies to make the practice session meaningful to the client was rated lowest (mean 2.5 at Time 1, 3.25 at Time 2) at both time points. Table 4 presents the p values of Wilcoxon analysis of change in CSR from Time 1 to Time 2.

### Behaviour

**Fig 3:**
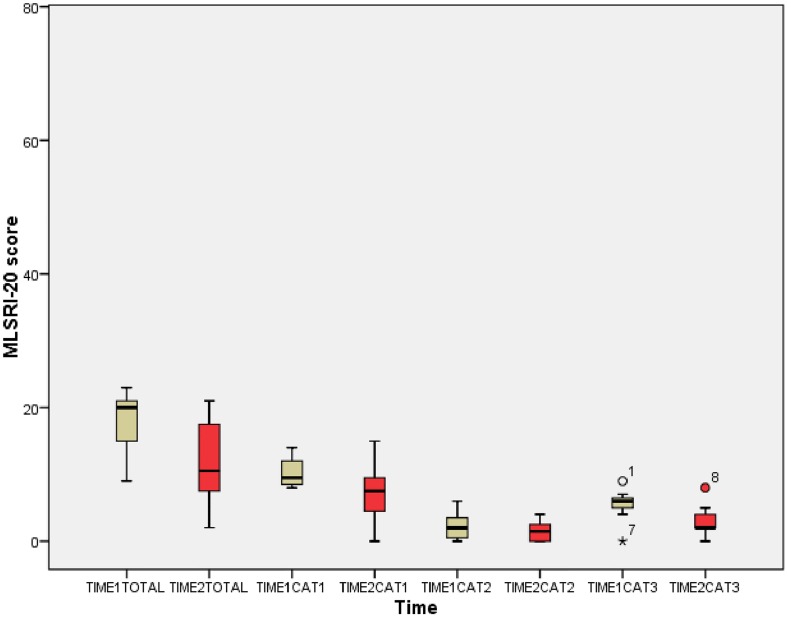
MLSRI-20 scores at Time 1 and Time 2.

Eight of the 11 therapists completed the MLSRI-20 at both time points. Mean overall scores at both Time 1 (18.8/76 = 24.7%) and Time 2 (11.8/76 = 15.5%) indicate a low observer-rated use of MLS. Fig 3 illustrates that no significant changes in MLSRI-20 total score (p = 0.092) or category scores (What Therapist Says: p = 0.173; What Therapist Does: p = 0.414; Practice: p = 0.051) were noted at Time 2. Table 5 summarizes the MLS that were observed to be used most and least frequently (i.e. the mode of each MLSRI-20 item) at each time point.

**Table 5:** Motor Learning Strategy Rating Instrument-20 Modes at Time 1 and Time 2.

Mode	Time 1	Time 2
4	None	None
3	None	None
2	Therapist provides encouragement Therapist directs attention externally Therapist directs attention internally Therapist verbalizes feedback relating to movement Therapist encourages client to learn from errors Practice is repetitive Practice is variable	Therapist directs attention internally Therapist verbalizes feedback relating to movement Practice is repetitive
1	Therapist verbalizes feedback about goal Therapist verbalizes feedback about success.	Therapist provides encouragement Therapist directs attention externally
0	Therapists asks rather than tells Therapists verbalizes about what was improved Therapist links to other activities Therapist encourages mental practice Therapist uses modelling Therapist uses guidance Therapist encourages practice outside of therapy Therapist provides education to a caregiver Practice is transferred to real life activities Practice progresses in difficulty	Therapist verbalizes feedback about goal Therapist verbalizes feedback about success. Therapists asks rather than tells Therapists verbalizes about what was improved Therapist links to other activities Therapist encourages mental practice Therapist uses modelling Therapist uses guidance Therapist encourages practice outside of therapy Therapist provides education to a caregiver Practice is transferred to real life activities Practice progresses in difficulty

### Focus groups

The following two identified themes from focus group data analysis were determined to relate to integrating a motor learning approach to IREX use.

1. Knowledge about MLS carried over to traditional interventions.

Therapists felt that the knowledge about MLS was applicable to other aspects of therapy and that they were familiar with this knowledge, but indicated that the content provided during the KT intervention provided a good refresher. Therapists were clear that the MLS information provided during the intervention carried over to their conventional treatments. For example, one therapist stated:“I thought it was great for me to reflect on the principles…it was really great to stop and think again, what kind of feedback am I giving and how, and just using—reapplying, I guess, the general principles that we had gone through to the IREX stuff for sure, but it also carried over to my regular treatment, too.”

2. Therapists focused preferentially on the MLS of transferring the skills focused on during IREX use to real world tasks.

Therapists reported that since the MLS knowledge felt so integral to their practice, they did not focus specifically on integrating all the MLS reviewed during the KT intervention during VR-based therapy. Instead, they focused on the strategy that was most relevant to the novelty of the VR approach: that of making sure tasks practiced during VR-based therapy carried over to functional improvements in real world activities. For example, one therapist said: “I needed to make a conscientious effort to have what we were doing with the IREX carry over to the functional task, like, to be pointing those things out, you know, like “oh you see, this could carry over to getting on the bus” or something… I needed to do that.” Therapists began by speaking to the clients to help them appreciate the practicality of participating in VR and by explaining to clients why they were using VR and how it was related to their functional goals. A therapist remarked: “I found myself doing a lot of that kind of talk prior to the session, sort of priming towards, “okay we’re going to go over—we’re going to try this, because I think you need to be able to learn this type of skill to be able to help you… navigating in this situation or doing this or that.”

## Discussion

This is the first study to evaluate the effectiveness of a KT intervention designed to enhance a motor learning approach to VR-based therapy in stroke rehabilitation. The MLS knowledge was provided in tandem with education about the VR system and its operation. This information was presented as theoretical and practical knowledge integral to VR system use.

Therapists reported improvements in MLS confidence, which were sustained at the end of the study. However, there was no change in clinical reasoning related to MLS use or in observed MLS use following practice using the IREX in clinical practice. While therapists stated in the focus group that they focused on transferring skills practiced during IREX sessions to real-life activities, this MLS was infrequently observed in the videotaped sessions, with a mode of 0 at both time points on the MLSRI-20. The MLSRI-20 rater was looking for evidence that therapists had verbally or physically made links to related tasks or activities in real life or had actually practiced related skills or tasks during the videotaped session. For example, if weight-shifting was practiced in an IREX game, then the therapist might include a real-life balance task or gait practice during the videotaped session. Instead of using this approach, it may well be that therapists focused on transfer during their usual, non-VR sessions, and that this demonstration of MLS use was not captured by the video recording. Indeed, the IREX sessions took place in a small treatment room that had some traditional therapy equipment, but lacked the space and the range of equipment of a usual gym. The KT intervention had presented information on how to integrate conventional therapy within PT or OT sessions that took place in the VR room, but therapists may have focused only on IREX use during their videotaped session. These treatment decisions likely had an impact on the ability of the MLSRI-20 to capture use of this particular MLS, which may explain the discrepancy between therapist self-report and observer ratings.

The lack of change observed with practice may have been related to the extent of information presented within the KT intervention. Therapists were expected to become proficient at understanding the VR system, its games and its technical operation [26] in addition to reviewing MLS knowledge and adapting MLS use to this approach. The KT intervention did not include the opportunity to practice using a motor learning VR approach with a standardized client prior to implementing the IREX with therapists’ own clients. Although the KT intervention did include case scenarios and practice with peers, these strategies may not have offered sufficient practice and feedback to allow therapists to understand how to apply MLS to this unique therapy approach. A greater opportunity to gain confidence in using the system before videotaping and outcome measurement may have enhanced therapists’ MLS integration, as well as expanded the range of clients from their caseloads with whom they felt comfortable recruiting into the study. This would have expanded the range of MLS that might be used and observed. Indeed, only 55% of therapists used the IREX with four clients as intended. For the others, there may not have been sufficient additional practice opportunities between Time 1 and Time 2 to elicit greater MLS application. Short client length of stay, challenges with a malfunctioning VR system leading to treatment delays, and reluctance of clients to participate in VR-based interventions had an impact on study recruitment. As all of these factors represent real-world clinical practice conditions, it is important to continue developing an improved understanding of best KT strategies in these types of contexts.

Although therapists indicated in the focus group that they felt MLS were integral to their usual practice, their pre-module confidence ratings were low. In a recent survey of Canadian physiotherapists in neurorehabilitation, 39.7% of respondents felt that they possessed only little to moderate knowledge of motor learning principles, although 51% felt confident or very confident in applying motor learning principles in practice [7]. Previous studies with PTs and OTs in differing practice settings have indicated that MLS are viewed as inherent to clinical practice and not something that therapists generally make explicit in their decision-making and clinical reasoning [41,42]. Therapists may have had difficulty articulating during CSR interviews how or why they were doing what they do in therapy from a motor learning perspective. It is important to note that these therapists had differing training backgrounds with respect to MLS education. A baseline test of motor learning knowledge would have elicited each therapist’s starting point. In addition, future directions for research could include evaluating MLS use during conventional therapy as a baseline from which to compare. Videotaping decisions were made in the context of burden on participating therapists and clients; indeed, the potential for anxiety related to being videotaped altering therapist behaviour (ref from the psychology lit would be an asset here) is a limitation of use of the MLSRI-20.

VR system attributes that likely influence therapist behavior may also explain the lack of MLSs observed. Therapists may have spent less time interacting with clients during the VR session because the client’s attention is required to be focused on the virtual environment. Indeed, MLS involving therapist hands-on interaction, such as modelling and guidance, were infrequently observed. Therapists did verbalize with clients, using the MLS of directing attention and of providing feedback about movement. This observation may indicate that therapists were verbally correcting movement patterns. Indeed, one of the common disadvantages of VR use is the potential for poor movement patterns to be elicited, given that users may be more motivated to achieve game success rather than to perform movements correctly [22,43]. Therapist instructions and feedback were amongst the MLS seen ‘often’ (a mode of 2 on the MLSRI-20). The motor learning literature suggests that augmented feedback should generally be presented less frequently over time, in order to avoid becoming dependent on therapist feedback and guidance [44], although whether this hypothesis holds true in acute stroke rehabilitation is unclear. However, it is important to note that the IREX provides feedback in the form of knowledge of results and knowledge of performance both during the task and subsequent to the task. During the focus groups, therapists did not discuss whether the information coming from the IREX influenced their own provision of feedback.

The outcome instruments did not measure the motor learning attributes afforded by the VR system itself, such as the feedback provided, or the client’s self-reported motivation to practice the VR tasks. One of the MLS that was observed ‘often’ at both time points was repetitive practice. VR systems may make increasing practice dosage feasible given the opportunity to set trial duration and to repeat tasks under the same conditions. Little is known about the extent to which VR systems alone, in the absence of therapist guidance, can be used as motor learning tools. A recent scoping review exploring the use of VR to improve balance and mobility disorders post-stroke argued that more research into the specific effects of extrinsic feedback, variable practice and motivation is needed to understand how each contributes to the benefits afforded by VR [12]. Overall, a need exists to understand which motor learning attributes in VR are most effective for learning, and what their optimal dosing should be [12]. A Cochrane systematic review of the use of VR in stroke rehabilitation emphasized the need to determine the most relevant characteristics of VR that enhance outcomes [45].

Subsequent steps for research include revising the e-learning modules and hands-on workshops to include study videotapes as examples of MLS use, and as a starting point for discussion about increasing MLS application during VR interventions. The revised e-learning modules will be housed on www.VR4Rehab.com, a website currently under development, that will include accessible resources for therapists interested in integrated VR systems into their clinical practice. Implications for research include the need for authors of motor learning-based VR intervention studies to make explicit the motor learning attributes of the systems and the strategies used by therapists during the interventions [16]. Kleynen et al. [8] highlight the need for in-depth description of motor learning interventions in clinical studies to illustrate how MLSs are applied.

## Conclusions

Promoting client motor learning after stroke is an essential element of neurorehabilitation. VR-based therapy may alter therapists’ use of MLS given that the technology commands much of the patient’s attentional resources and already provides feedback about movement and results. Although MLS integration and clinical reasoning as measured using observer-rated outcome instruments following a KT intervention designed to promote MLS use in VR-based therapy did not increase, we report gains in motor learning knowledge by therapists that they judged to be relevant beyond their VR-based interventions. Understanding the differences in MLS application across various types of interventions can inform the content of future KT initiatives in this rapidly developing area of practice.

## Supporting Information

S1 Table De-identified results for each participant on the Motor Learning Strategy Rating Instrument, the Chart-Stimulated Recall, and the e-learning modules. (XLSX)
